# Spectroscopic and Microscopic Characterization of Microbial Biofouling on Aircraft Fuel Tanks

**DOI:** 10.1021/acs.langmuir.3c02803

**Published:** 2024-02-06

**Authors:** Jaime Gómez-Bolívar, Martin P. Warburton, Adam D. Mumford, Juan F. Mujica-Alarcón, Lorna Anguilano, Uchechukwu Onwukwe, James Barnes, Myrsini Chronopoulou, Yon Ju-Nam, Steven F. Thornton, Stephen A. Rolfe, Jesús J. Ojeda

**Affiliations:** † School of Biosciences, University of Sheffield, Sheffield S10 2TN, U.K.; ‡ Department of Chemical Engineering, Faculty of Science and Engineering, Swansea University, Swansea SA1 8EN, U.K.; § Experimental Techniques Centre, Brunel University London, Uxbridge UB8 3PH, U.K.; ∥ Airbus Operations Ltd, Pegasus House, Aerospace Avenue, Filton, Bristol BS34 7PA, U.K.; ⊥ Conidia Bioscience Ltd, Bakeham Lane, Englefield Green, Egham TW20 9TY, U.K.; # Groundwater Protection and Restoration Group, Department of Civil & Structural Engineering, Broad Lane, University of Sheffield, Sheffield S3 7HQ, U.K.

## Abstract

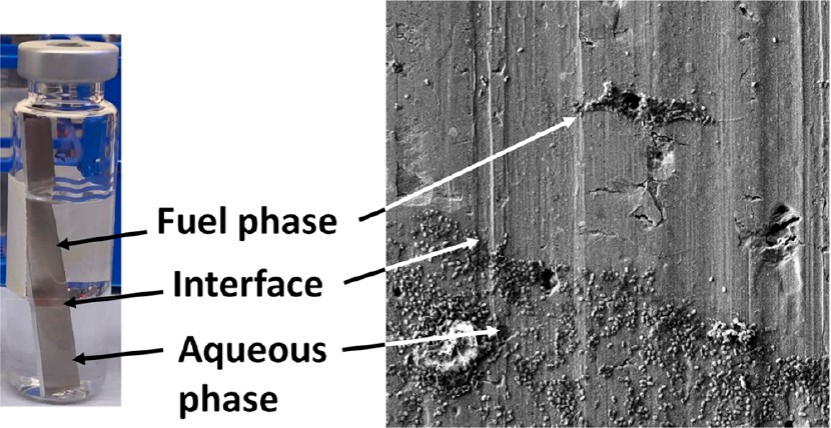

Avoiding microbial contamination and biofilm formation on the surfaces of aircraft fuel tanks is a major challenge in the aviation industry. The inevitable presence of water in fuel systems and nutrients provided by the fuel makes an ideal environment for bacteria, fungi, and yeast to grow. Understanding how microbes grow on different fuel tank materials is the first step to control biofilm formation in aviation fuel systems. In this study, biofilms of *Pseudomonas putida*, a model Gram-negative bacterium previously found in aircraft fuel tanks, were characterized on aluminum 7075-T6 surfaces, which is an alloy used by the aviation industry due to favorable properties including high strength and fatigue resistance. Scanning electron microscopy (SEM) coupled with energy-dispersive X-ray (EDX) showed that extracellular polymeric substances (EPS) produced by *P. putida* were important components of biofilms with a likely role in biofilm stability and adhesion to the surfaces. EDX analysis showed that the proportion of phosphorus with respect to nitrogen is higher in the EPS than in the bacterial cells. Additionally, different morphologies in biofilm formation were observed in the fuel phase compared to the water phase. Micro-Fourier transform infrared spectroscopy (micro-FTIR) analysis suggested that phosphoryl and carboxyl functional groups are fundamental for the irreversible attachment between the EPS of bacteria and the aluminum surface, by the formation of hydrogen bonds and inner-sphere complexes between the macromolecules and the aluminum surface. Based on the hypothesis that nucleic acids (particularly DNA) are an important component of EPS in *P. putida* biofilms, the impact of degrading extracellular DNA was tested. Treatment with the enzyme DNase I affected both water and fuel phase biofilms—with the cell structure disrupted in the aqueous phase, but cells remained attached to the aluminum coupons.

## Introduction

Microbial communities can contaminate fuel systems using the fuel as a source of carbon and nutrients to sustain their growth in the condensed water found in fuel tanks, at the fuel–water interface, in the fuel itself, or as biofilms on surfaces. The hydrocarbons in jet fuel provide an abundant energy source to those microbes that are able to metabolize them, and fuel also contains other trace nutrients to sustain microbial growth.^1^ The presence of water is essential for active growth.^2^

Water can enter aviation fuel systems as dispersed water droplets (less than 40 μm) in the fuel (although the allowable limits are low at 30 ppm), as water vapor from the environment (especially in humid regions), which then condenses, or as rainwater seeping into tanks during filling.^1^ Water can exist in three states inside the fuel tank: dissolved, free water in suspension, and settled water. Water is soluble in jet fuels up to approximately 60 ppm at 25 °C, although the presence of microbes can modify these levels.^1,3,4^ Water can emulsify by dispersing as tiny suspended droplets, which can coalesce and settle out onto the tank surfaces.

Temperatures in the tank vary significantly. During flight, temperatures can reach as low as −47 °C, causing all available free water to freeze.^5,6^ However, during taxiing, parking, and particularly overnight storage, water temperatures can reach ambient conditions, producing conditions amenable to microbial growth and biofilm formation. The most extreme conditions are found in the tank’s extremities, where temperatures can fluctuate between +40 and −47 °C. Despite this hostile environment, microbes are able to grow wherever there is available water. The aviation industry must contend with microorganisms contaminating jet fuel systems and biofilm growth on tank surfaces.

Microorganisms use enzymes such as dehydrogenases, lactases, and peroxidases to oxidize and degrade alkenes, alkanes, and aromatic compounds present in aviation fuels and, by metabolite excretion, can alter the fuel chemistry and cause its deterioration.^7−9^ Microbes also cause biofouling of fuel system components such as pipelines and filters, including microbially induced corrosion (MIC) of storage tanks and the protective paints on tank surfaces.^10−13^ Microbes can lead to MIC by various routes, including the release of protons and organic acids (for example, acetic, formic, oxalic, citric, or carbonic acid) as a consequence of hydrocarbon degradation.^14−16^

Biofilms are composed of cells and extracellular polymeric substances (EPS), with the latter accounting for approximately 50–90% of the total organic carbon.^17^ The EPS comprises a conglomeration of different biopolymers, forming a three-dimensional biofilm architecture, and contributes to adhesion to surfaces and cohesion of biofilm members.^18^ Additional surface-associated adhesion molecules, including those in flagella and pili, have been demonstrated to play an important role in binding to abiotic substrates and other bacteria, thereby leading to surface colonization and biofilm formation.^19^

Understanding the physicochemical properties of microbes and associated EPS is important to identifying the processes that govern microbial attachment to surfaces inside the fuel tanks. EPS composition in bacterial biofilms is complex and depends on the microbial strain and environmental conditions in which the biofilms develop, such as the concentration of nutrients, hydrodynamic conditions, and bacterial mobility, among other factors. These EPS consist mainly of polysaccharides, proteins, lipids, and nucleic acids.^18,20^ EPS are hypothesized to play an important role in bacterial attachment,^17,18,21−23^ and the molecules involved in the adhesion and biofilm formation depend on nutrient availability and the physicochemical properties of the surface (for example, hydrophobicity, charge, and surface roughness).^24^

In the current study, biofilms of the model organism *Pseudomonas putida*, commonly found in the fuel tanks of aircraft,^25^ were characterized on aluminum alloy AA7075-T6, used in aircraft fuel tanks due to properties such as high strength and fatigue resistance. *P. putida*’s capacity to utilize aviation fuel as the sole carbon source has been observed previously.^25^ However, there is limited information about the mechanisms of biofilm formation and the specific macromolecules engaged in this process in aircraft materials. *P. putida*’s effectiveness in hydrocarbon degradation has been demonstrated as a sustainable approach for soil decontamination polluted with hydrocarbon.^26^ However, it has also been identified as a significant challenge in the aeronautical industry, particularly for the contamination observed on aircraft fuel tanks.^25^ A combination of spectroscopic and microscopic techniques (such as confocal microscopy, electron microscopy, epifluorescence microscopy, and micro-Fourier transform infrared spectroscopy (micro-FTIR)) were used to determine which macromolecules are involved in the attachment of bacteria to surfaces in the water phase, in the fuel phase, and at the water–fuel interface. By using this multidisciplinary approach, a deeper understanding of the chemical composition of the EPS within the biofilm and the influence of specific extracellular molecules on the biofilm adhesion to the aluminum surface, grown in the fuel and water phase, can be obtained.

## Materials and Methods

### Microcosm Design and Inoculum

*Pseudomonas putida* F1 (ATCC 700007) cells were grown in LB medium, washed, and resuspended in 1/4-strength Bushnell-Haas medium to an optical density of 0.05. Microcosms were prepared by placing an aluminum coupon (63 × 10 × 1 mm^3^) in a 20 mL glass vial containing 7 mL of quarter-strength Bushnell-Haas medium with cells and 7 mL of Merox treated Jet A-1 aviation fuel. The fuel was passed through Attapalgus clay (Fuller’s Earth) to remove contaminants and additives and then filter-sterilized by passing it through a 0.22 μm nitrocellulose filter (Whatman, USA). The Attapalgus clay was obtained from a filter (CO-718CE Parker Velcon CO Series Clay Canister Cartridge) that is used commercially to treat fuel as it is loaded into aircraft. All other materials were autoclaved before use. Microcosms were incubated at 25 °C for either 4 or 8 weeks.

### Studies of Biofilm Coverage Using Epifluorescence Microscopy

Coupons with the attached microbial communities were fixed in 1% w/v formaldehyde, and cells were stained with 1:800 diluted SYTO9 (5 mM) (Thermofisher, UK). The coupons were imaged with an epifluorescence microscope (Leica DM6) using blue light excitation (470 nm), and the emission was detected at 510 nm. Images were taken by using a 10× objective. As the coupons are not optically flat, images were taken at different focal planes, and an extended focus image was produced by maximum intensity projection in the *Z*-axis. The images were analyzed in ImageJ.^27^ Thresholds were set to identify microbes, and the percent surface area covered by the cells was calculated.

Fifteen images were taken of each coupon. The fuel–water interface was located, and 3 images were taken before additional sets of 3 images at 5 and 10 mm into the fuel and aqueous phases, respectively. Three biological replicates were prepared.

### Confocal Microscopy Analysis with Specific Dyes

The location of specific compounds within biofilms was determined with compound-specific dyes and confocal microscopy using an Olympus FV1000 confocal microscope and a 60× objective, with a Z step size of 0.1 μm. SYTO-9 was used to stain total DNA (both genomic and extracellular, 488 nm argon laser λ_ex_ 488 nm, λ_em_ 515 nm). BOBO-3 (1:800 diluted 5 mM) was used to stain extracellular DNA, and ConA-tetramethylrhodamine (ConA-Rho) was used to stain polysaccharides (200 μg/mL in 0.1 M sodium bicarbonate solution). Both were excited by using a 514 nm HeNe laser. Lipids were stained with Nile Red (100 μL of a 0.05 mg/mL stock solution diluted in 900 μL of acetone) with detection using the lambda scan option with a 20 nm bandwidth from 550 to 700 nm.

### Scanning Electron Microscopy Images

Scanning electron microscopy (SEM) images were obtained using a Zeiss Supra 35VP high-resolution scanning electron microscope at variable pressure (Environmental SEM), equipped with a field emission gun and an energy dispersive X-ray spectroscopy (EDX) facility.

### Micro-FTIR Analysis

Micro-FTIR images were obtained using a PerkinElmer Spotlight micro-FTIR spectroscope, equipped with a mercury–cadmium–telluride detector (consisting of 16 gold-wired infrared detector elements). A per-pixel aperture size of 25 μm × 25 μm was used with two coadded scans per pixel and a spectral resolution of 16 cm^–1^.

### DNase Treatment of *P. putida* Biofilm on AA7075-T6

For DNase treatment, *P. putida* was grown as described above (microcosm on AA7075-T6 incubated for 4 weeks). After 4 weeks incubation coupons with attached microbial communities were incubated in humid air for 15–30 min to allow the fuel vapor to evaporate. DNase I at a concentration of 270 U/mL (ITW reagents) was added into a 10 mL Falcon tube containing DNase I buffer with the coupon and then incubated at 37 °C for 48 h. For controls, distilled water was added instead of DNase I. After incubation with DNase I, the liquid phase was removed, coupons were washed four times with 0.9% (w/v) NaCl, and the cells were stained with SYTO-9.

## Results and Discussion

### Biofilm Coverage on AA7075-T6 in the Water and Fuel Phases

**Figure 1:**
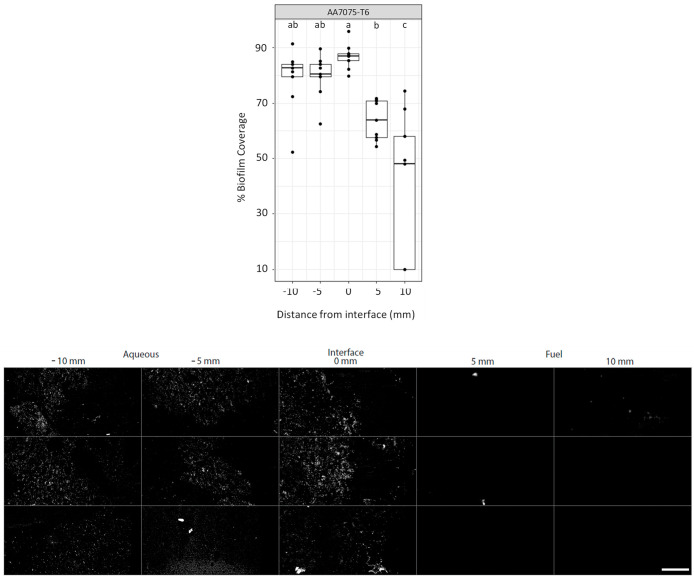
Top: proportion of surface covered by *P. putida* biofilm after 4 weeks of growth on AA7075-T6. Measurements were taken in the aqueous phase (−10 and −5 mm), at the aqueous–fuel interface (0 mm), and in the fuel phase (5 and 10 mm). The percentage of coverage for fields of view were measured in triplicate for each of the three biological replicates. Results are shown as boxplots, with the mean and interquartile ranges. Individual measurement values are shown as points. Samples that share a letter did not differ significantly from each other. Bottom: examples of epifluorescent microscopy images of *P. putida* biofilms on AA7075-T6 were used to assess the proportion of biofilm coverage. The scale bar is 10 μm.

An initial survey of *P. putida* attachment after 4 weeks of incubation showed marked variation in biofilm coverage depending on the phase the biofilm developed in as shown in Figure 1.

On the AA7075-T6 coupons, biofilm coverage was greatest in the aqueous phase and at the aqueous–fuel interface. Biofilm coverage in the fuel phase was markedly lower and decreased as the distance to the interface increased.

### Confocal Microscopy of *P. putida* Biofilms on Aluminum Alloy AA7075-T6

**Figure 2:**
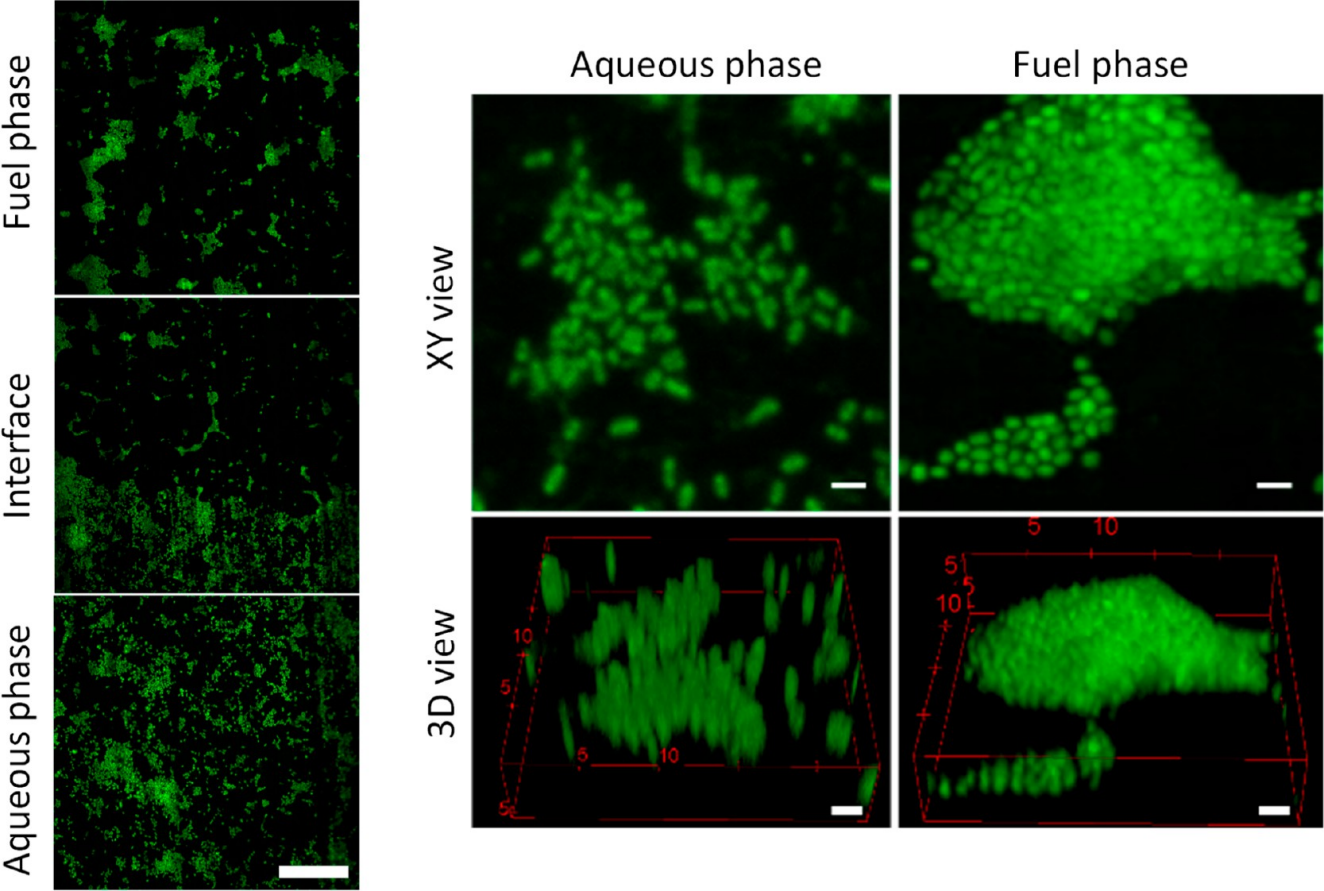
Left: *P. putida* biofilm formation in the aqueous, fuel–water interface, and fuel phases after 8 weeks growth on AA7075-T6. Cells were stained by using SYTO-9. Scale bar: 50 μm. Right: images of *P. putida* biofilm after 8 weeks of growth on AA7075-T6, showing different morphologies in the aqueous and fuel phases. Scale bar = 2 μm.

To better understand the differences in biofilm attachment, *P. putida* biofilms were allowed to develop longer, for 8 weeks, on AA7075-T6 coupons and then imaged using confocal microscopy (Figure 2). Samples were stained using the DNA-specific dye, SYTO-9. Biofilm coverage was greater in the aqueous phase and at the aqueous–fuel interface than in the fuel phase, in agreement with the observations in the previous section. The morphology of the biofilm differed between the aqueous and fuel phase. Cells in the aqueous phase were separate and more uniformly distributed across the surface, while localized clusters or aggregations were observed in the fuel phase. The agglomerated morphology of *P. putida* biofilms in the fuel phase, compared to separate cells in the aqueous phase, is also shown in Figure 2. In addition to the bright staining of DNA in individual cells, very diffuse background staining could be seen.

**Figure 3:**
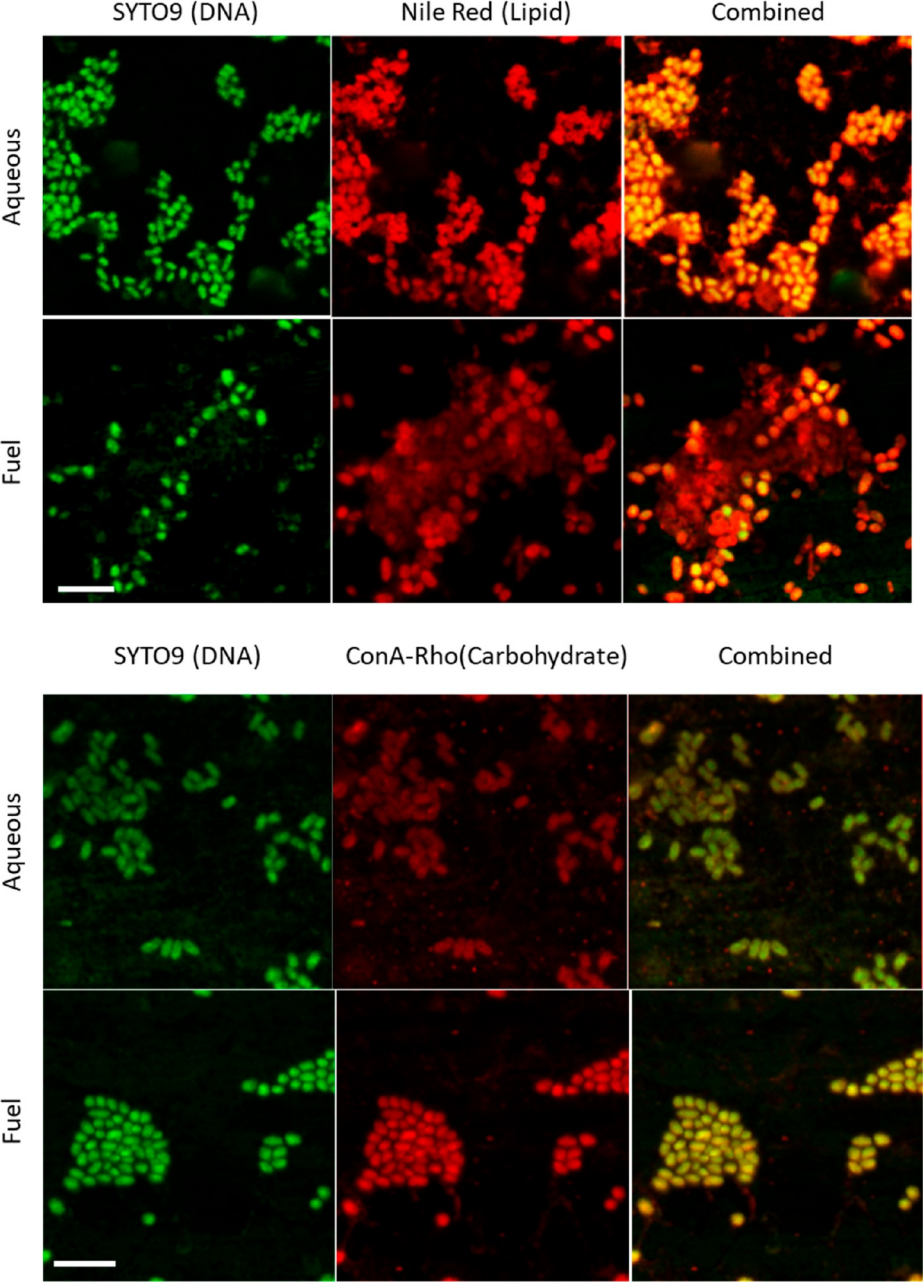
Staining of *P. putida* biofilms on AA7075-T6, in the aqueous and fuel phases. DNA was stained with SYTO-9, lipids with Nile Red, and polysaccharides with ConA-Rho. Scale bar: 5 μm.

Figure 3 shows the biofilm grown on AA7075-T6 alloy stained with Nile Red (lipids), ConA-Rho (carbohydrates), and SYTO-9 (DNA). In the aqueous phase lipids were closely associated with the cells, but in the fuel phase they were distributed more widely across the aluminum surface, joining different cells together. Polysaccharides and DNA were present, both associated with cells and also on the aluminum surface.

**Figure 4:**
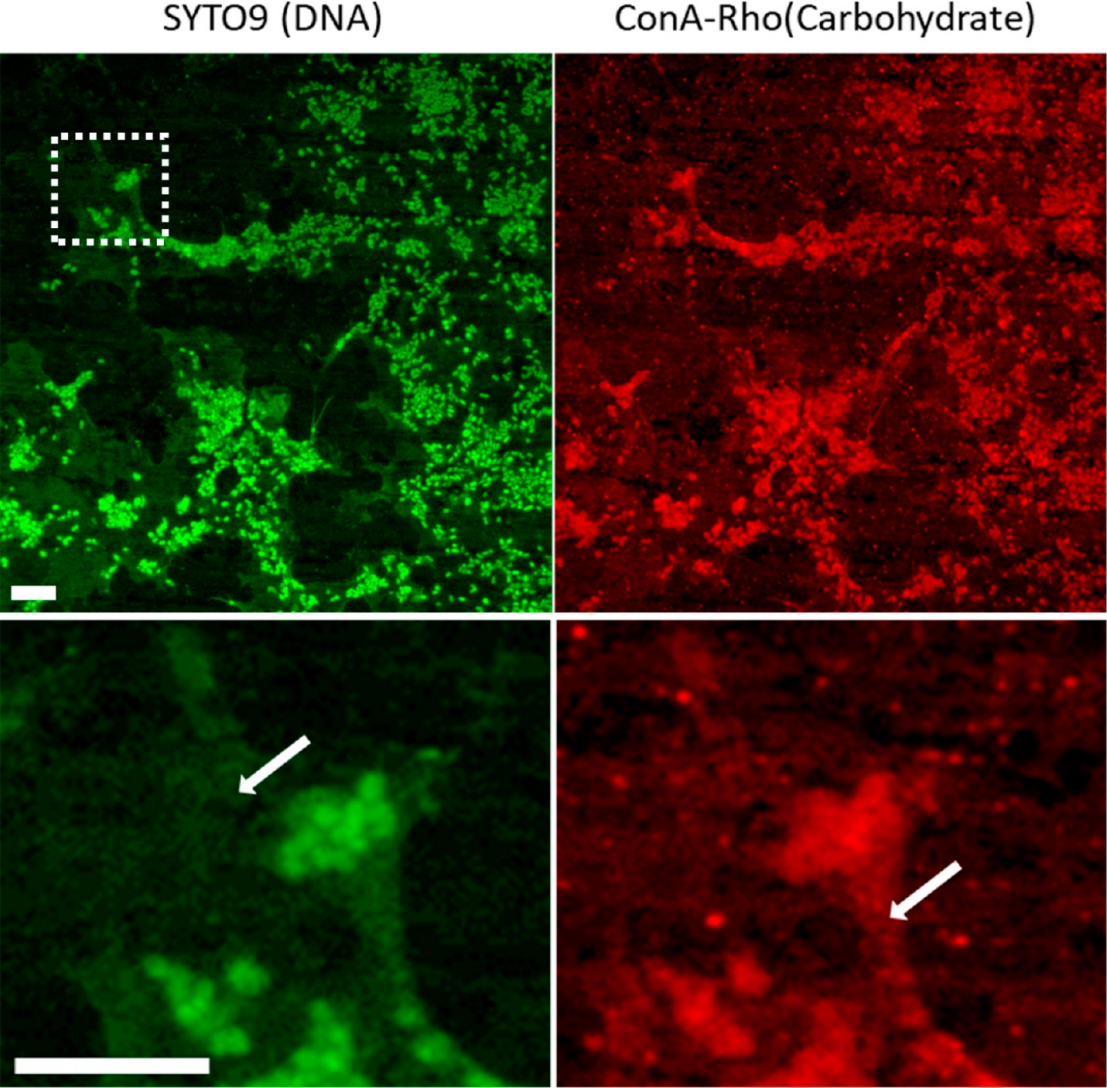
Staining of DNA (SYTO 9 green) and polysaccharide (ConA-Rho Red) in *P. putida* biofilms grown on AA7075-T6 in the fuel phase. Arrows indicate regions where DNA and polysaccharides are located away from the cells. Scale bars = 10 μm.

Figure 4 shows more clearly the widespread presence of both DNA and polysaccharides in biofilms in the fuel phase on aluminum AA7075-T6. These macromolecules were present associated with cells and also away from cells, indicating their presence in the biofilm matrix.

Confocal microscopy using molecule-specific dyes suggested that the attachment of *P. putida* biofilms on the AA7075-T3 is mediated by excreted EPS, particularly in the fuel phase. The EPS keeps the cells together, forming localized cell aggregates or clusters in the fuel phase. These adhere to the aluminum surface and could provide physical stability and protection of the biofilm. The presence of polysaccharides, lipids, and DNA outside the cells on the aluminum surfaces indicates that these macromolecules are major constituents of the EPS. The hydrophobic nature of lipids could allow water retention within the internal channels of the biofilm, vital in the hydrophobic fuel phase, while facilitating access and sharing of limited nutrients. This lipid-rich phase would act as a barrier between the cells and the fuel, trapping a hydrophilic environment within the cluster of cells on the aluminum surface.

### Scanning Electron Microscopy Analysis of *P. putida* Biofilm

**Figure 5:**
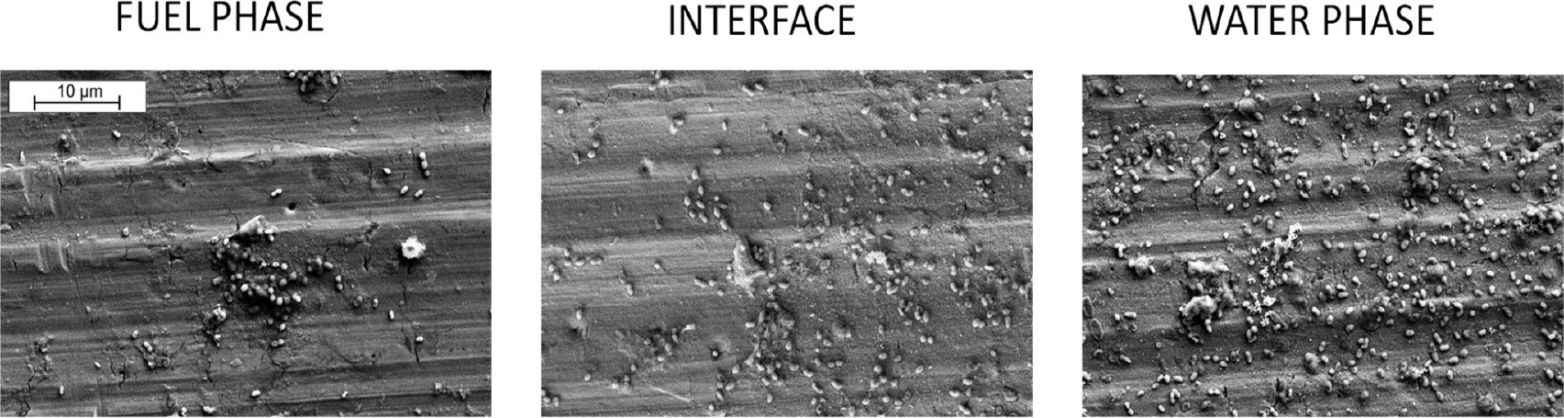
SEM micrographs of biofilm formation of *P. putida* on AA7075-T6, showing different morphologies in the fuel and water phases. While the cell distribution in the water phase seems to be more uniform, cells at the fuel phase tend to form aggregates or clusters. Scale bar represents 10 μm.

Cell aggregates and clusters, joined together by EPS, were evident in the fuel phase for biofilms grown on AA7075-T6 (Figure 5), supporting the results obtained using confocal microscopy. In the aqueous phase, bacteria showed less EPS production and were more uniformly dispersed on the aluminum surfaces.

**Figure 6:**
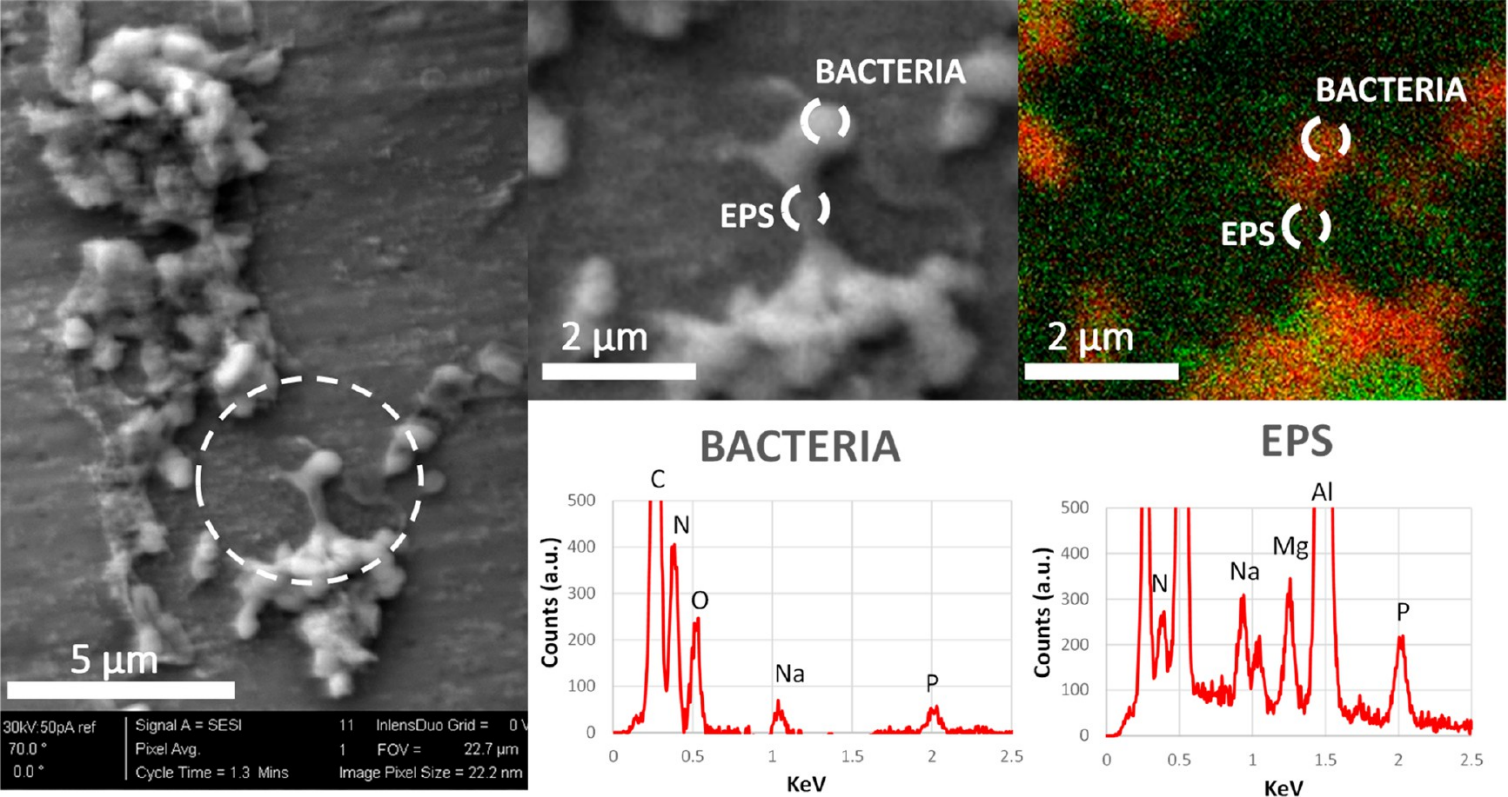
SEM micrographs combining EDX elemental mapping of the relative distribution of nitrogen (N) and phosphorus (P) in a *P. putida* biofilm on AA7075-T6 grown in the fuel phase. Left: SEM micrograph of biofilm in the fuel phase. Center: regions selected for elemental mapping using EDX. Right: relative distribution of N and P across the biofilm. Areas where N is more abundant are shown in red, and areas where P is more abundant are shown in green. Al and Mg peaks come from the AA7076-T6 composition.

While confocal microscopy provides information at the macromolecular level of the main biofilm components, SEM/EDX mapping can provide spatial distribution and semiquantitative information on the elements present. Figure 6 shows the results of SEM/EDX analysis of *P. putida* and the associated EPS on AA7075-T6 in the fuel phase.

Elemental mapping showed that C, N, O, and P were the main constituents of both bacteria and EPS, but with different proportions in each case. The proportion of N with respect to P is much higher in the EDX spectrum of the bacterial cell when compared to the EPS. Al and Mg peaks come from the AA7076-T6 composition due to the proximity of the EPS to the surface. Nitrogen and phosphorus were distributed heterogeneously across the biofilm, with EPS having a higher concentration of P-containing molecules (e.g., molecules such as DNA and phospholipids). In addition to DNA, phosphorus-containing molecules such as phospholipids and glycerophospholipids may be important components of EPS, as it has been previously found that some strains of *P. putida* can secrete these macromolecules.^28^

### Micro-FTIR Analysis of *P. putida* Biofilms on AA7075-T6 across the Water and Fuel Phases

**Figure 7:**
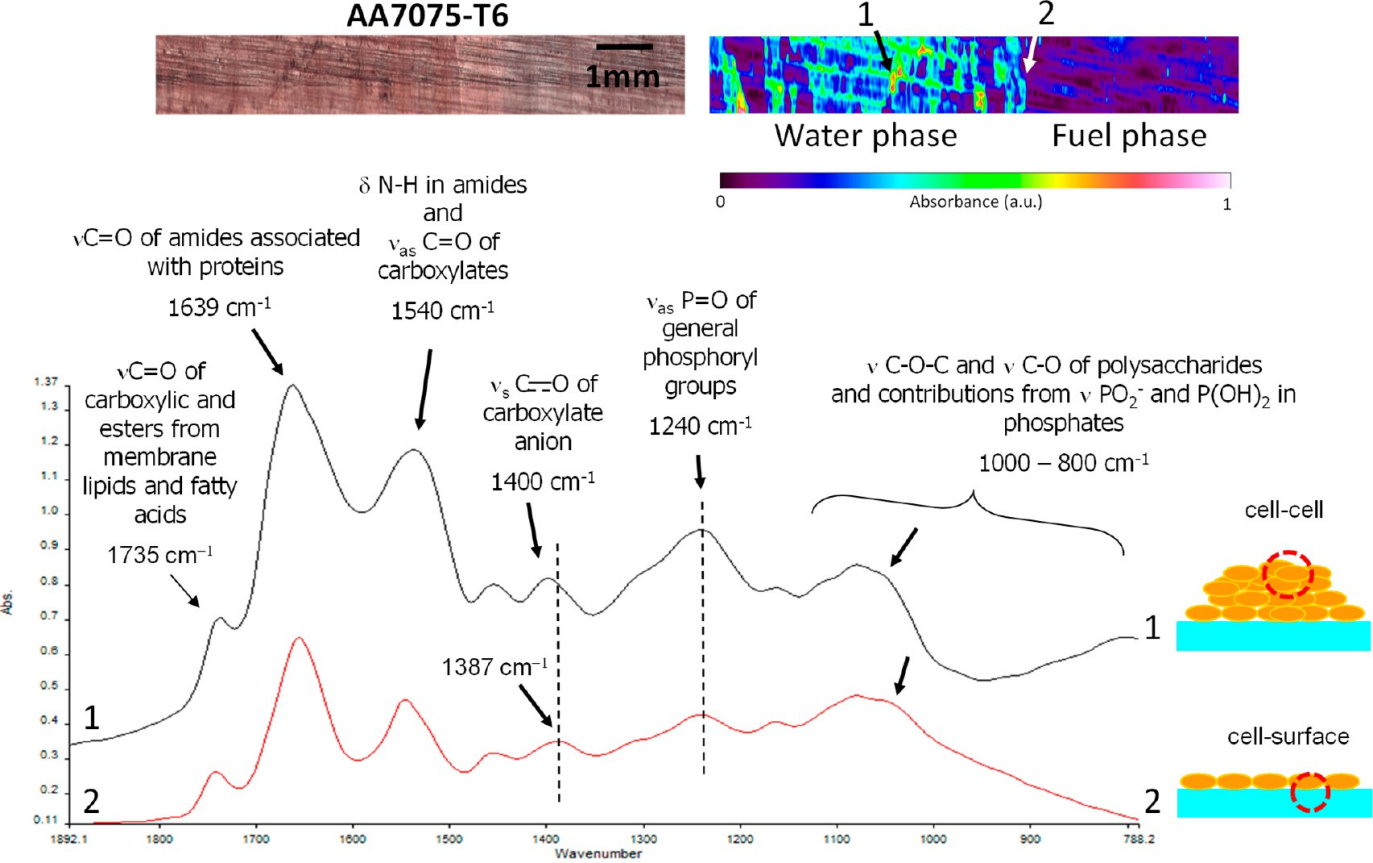
Reflectance micro-FTIR spectroscopy for the study of *P. putida* attached to AA7075-T6. False-color images showing the presence and abundance of IR-absorbing molecules on the surface of the aluminum coupon. Spectrum 1 corresponds to a region with “thick” or highly abundant agglomeration of cells, whereas spectrum 2 corresponds to an area where the absorbance intensity in the false-color image was much weaker, indicating less presence of cells or a much thinner biofilm.

The FTIR spectra of *P. putida* adhered to AA7075-T6 were analyzed to evaluate the influence that organic functional groups may have on the mechanism of adhesion onto the metal surface. The results obtained from the micro-FTIR experiments confirmed the presence of hydrogen-, carbon-, nitrogen-, and phosphorus-containing macromolecules within the biofilms. Blue areas on the false color image gave a flat spectrum (no IR-absorbing bands) and are likely to correspond to the aluminum surface only, without detectable biomass. Typical wavelength ranges and correlated functional groups in FTIR spectra are presented for *P. putida* in Figure 7.

The assignment of the functional groups and the corresponding frequencies for the *P. putida* spectra are summarized in Table 1. All the FTIR spectra of the analyzed biofilms were located around 1639 and 1540 cm^–1^, corresponding to the amide I and II bands, respectively. The amide I band is due to the stretching carbon–oxygen double bonds (νC=O) within amides associated with proteins, and the amide II band is a combination of bending N–H bonds (δN–H) within amides and contributions from stretching C–N (νC–N) groups. The peak at 1455 cm^–1^ also conceals the amine III group. The peak around 1400 cm^–1^ is due to the symmetric stretching C–O bond of carboxylate groups (ν_sym_COO^–^), while the asymmetric stretching (ν_asym_COO^–^) is concealed by the amine II band at 1540 cm^–1^. The signal around 1735 cm^–1^ is actually a combination of two peaks: a signal corresponding to the vibrational C=O stretching (νC=O) of carboxylic acids at 1735 cm^–1^ and another peak at 1725 cm^–1^ corresponding to the stretching C=O of ester functional groups from membrane lipids and fatty acids. The double-bond stretching of phosphate–oxygen ⟩P=O (νP=O), which is connected to phosphodiesters of nucleic acids and general phosphoryl groups, is observed in Figure 9 at 1240 cm^–1^. Vibrations of −COOH and C–O–H are located here as well. The peak at 1225 cm^–1^ within that band could indicate double-bond stretching of phosphates. Additionally, the stretching of P=O groups of polyphosphate products and phosphorylated proteins is present at around 1080 cm^–1^. The region between 1200 and 950 cm^–1^ shows the C–O–C and C–O–P stretching of diverse polysaccharide groups and is usually called the “polysaccharide region”.

**Table 1:** Infrared Absorption Bands of the *Pseudomonas putida* Functional Groups

wavenumber (cm^–1^)	functional group assignment^29−39^
1735	stretching C=O of ester functional groups from membrane lipids and fatty acids; stretching C=O of carboxylic acids
1639	stretching C=O in amides (amide I band)
1540	N–H bending and C–N stretching in amides (amide II band); asymmetric stretching for deprotonated COO^–^ groups
1400	symmetric stretching for deprotonated COO^–^ group
1455	bending CH_2_/CH_3_ (scissoring) and amide III group
1384	bending CH_2_/CH_3_
1305	vibration C–N from amides
1300–1250	vibrations of C–O from esters or carboxylic acids
1240	vibrations of −COOH and C–O–H; double-bond stretching of ⟩P=O of general phosphoryl groups and phosphodiester of nucleic acids
1225	stretching of P=O in phosphates
1200–950	asymmetric and symmetric stretching of PO_2_^–^ and P(OH)_2_ in phosphates; vibrations of C–OH, C–O–C, and C–C of polysaccharides
1080	stretching P=O of phosphodiester, phosphorylated proteins, or polyphosphate products
976	symmetric stretching vibration of phosphoryl groups

Considering the FTIR spectra in Figure 7, spectra 1 for the *P. putida*, corresponding to a region with “thick” or highly abundant agglomeration of cells, showed a shift in the peak due to the symmetric stretching of carboxylate groups to higher frequencies (1400 cm^–1^), when compared to the region with fewer cells, or a much thinner biofilm on the aluminum surface (1387 cm^–1^). These shifts have also been previously reported on *P. putida* biofilms growing on iron oxides^36^ and have also been observed on *Aquabacterium commune* biofilms (a Gram-negative bacteria) on stainless steel surfaces.^35^

**Figure 8:**
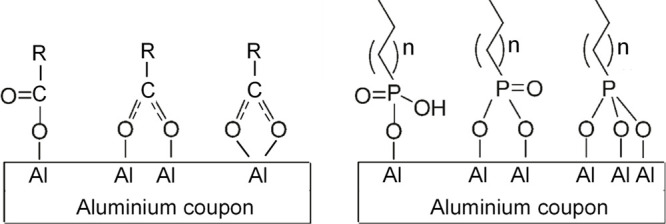
Metal complexes of carboxylate and phosphoryl groups. Left: bidentate, binuclear, and bidentate mononuclear carboxylate complexes. Right: unidentate, bidentate, and tridentate phosphoryl complexes.^41^

There have been several studies on metal complexes of carboxylic acids which have established an experimental correlation between the position of the symmetric and asymmetric stretching bonds of the carboxylate groups.^35,36,40^ The difference between the symmetric and asymmetric frequencies and the corresponding organic–metal complex (Figure 8) is denoted by Δ*v*. This difference is described in descending order as follows:
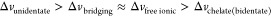
Unidentate complexes have a Δ*v* greater than 200 cm^–1^, and normally the symmetric stretching is lowered to a reduced frequency. The bidentate chelating has a Δ*v* less than 100 cm^–1^, and the symmetric stretching is shifted to higher frequencies, whereas asymmetric stretching is decreased to a lower frequency. For bridging complexes, the Δ*v* ≈ 160 cm^–1^, and the position of the symmetric and asymmetric can move in any direction.^40^

The spectra of the thinner *P. putida* biofilm had the symmetric stretching band shifted to a lower wavelength by about 12 cm^–1^. The large amide band at around 1540 cm^–1^ may be masking the asymmetric stretching of the carboxylate groups; however, no evident shoulders were observed near the amide II band, suggesting that the asymmetric stretching (ν_asym_COO^–^) of carboxylate groups might not have a significant shift to either lower or higher frequencies beyond the 1540 cm^–1^ wavenumber. From these results, it might be assumed that the carbonyl functional groups of microorganisms form bidentate and bridging complexes with the surface of the aluminum alloys.

In addition to the carboxylate bands in the FTIR spectra, it was also observed that there were significant differences in the range 1200–950 cm^–1^, a region that is normally attributed to phospholipids, indicating that these functional groups are also responsible for the irreversible covalent bond between the biofilm and the metal (see Figure 8).

From the FTIR spectra it could be presumed that the main moieties responsible for the attachment of *P. putida* to the aluminum alloys, in particular providing stability to the biofilm at the fuel phase, are carboxylic groups (or carboxylates) and phosphoryl groups. Given that the aperture of the FTIR microscope is 25 μm, it is not possible with this technique to distinguish between cells and EPS. However, combining this observation with the results obtained with confocal microscopy and SEM, it is likely that these bonds occur between secreted EPS molecules and the metal surface. EPS macromolecules containing carbonyl functional groups (arising from proteins and amino acids at the biofilm–metal interface) and phosphoryl groups (from nucleic acids and phospholipids) may form bidentate and bridging complexes with the surface of the aluminum alloy. It is important to highlight that adhesion proteins (adhesins), such as those found in pili, also contain carboxylic groups and could, therefore, contribute to the formation of carboxylate complexes similar to those described in Figure 8.

### Analysis of DNase Treated Samples of *P. putida* Biofilm on AA7075-T6

Based on the hypothesis that nucleic acids (e.g., DNA) are an important component of EPS in *P. putida* biofilms, the impact of degrading extracellular DNA was tested. Mature biofilms of *P. putida* grown on AA7075-T6 were exposed to deoxyribonuclease I (DNase I)—an enzyme that cleaves the phosphodiester bonds in the DNA backbone. Samples were stained with SYTO-9 after incubation with DNase I and analyzed using epifluorescence microscopy.

**Figure 9:**
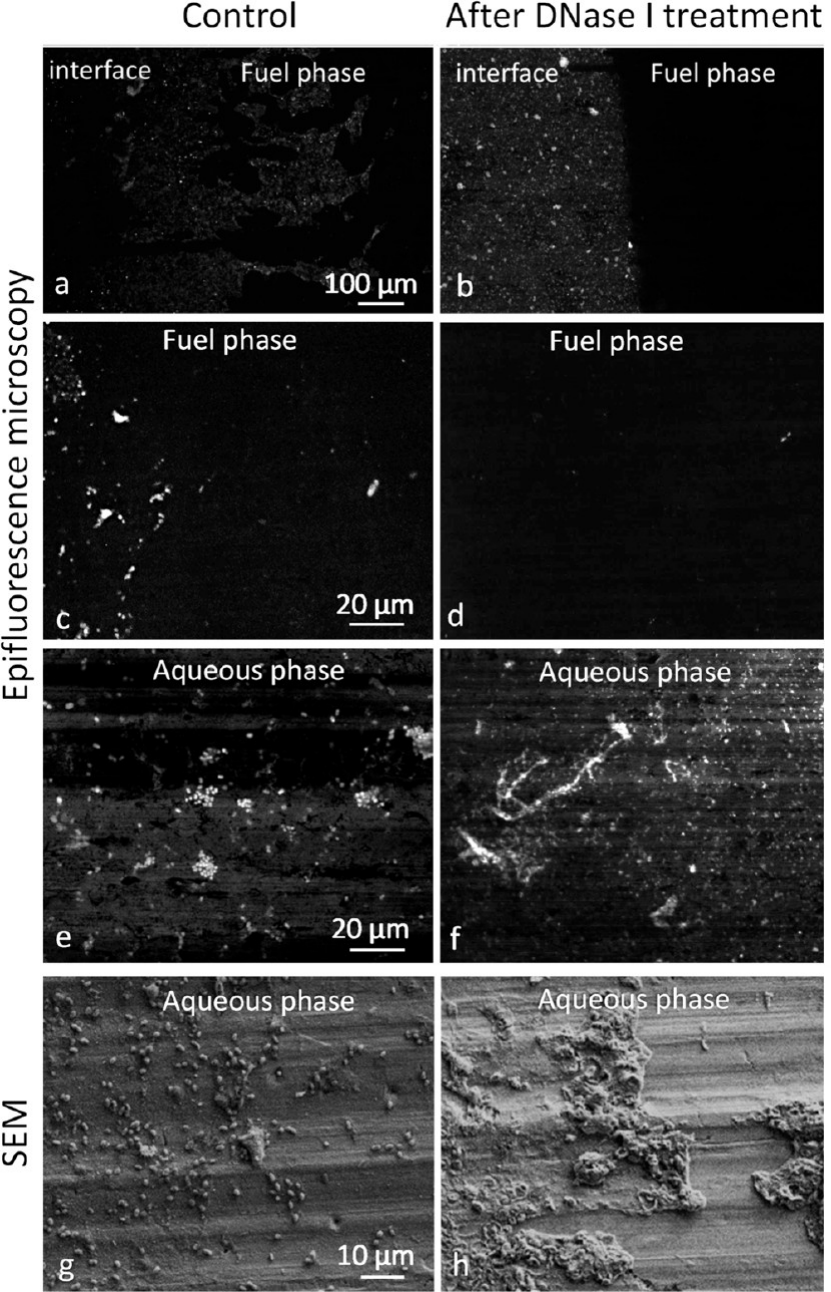
Epifluorescence images of *P. putida* growing on the AA7075-T6 water–fuel interface (a, b) and higher magnification at the fuel phase (c, d) and aqueous phase (e, f). Cells were stained with SYTO-9. Control without DNase I treatment (left) and DNase I treated biofilm (right). Bottom panels (g, h) show SEM images of *P. putida* at the aqueous phase without DNase I treatment (left) and DNase I treated biofilm (right).

Figure 9 shows that DNase I treatment impacted biofilms grown at both the aqueous and fuel phases. Epifluorescence microscopy images showed morphological differences between the controls and the DNase I treated biofilm. In the fuel phase, DNase I removed the biofilm, while in the aqueous phase it disrupted the biofilm structure, but biomass still remained adhered to the surface. Higher magnification images showed morphological differences between the controls and the DNase I treated biofilm, with the structure of the cells being disrupted but still attached to the coupon in the aqueous phase. Scanning electron microscopy images also confirmed this observation (see panels g and h in Figure 9). This means that although the enzymatic treatment disrupted the attached cells, cell debris and EPS residues were still present on the surface at the aqueous phase, and this could potentially constitute a suitable substrate for new cells to grow.

These results indicate that eDNA is an essential macromolecule for the attachment of the cells to the surface of AA7075-T6 in the aqueous phase. It also indicates the different EPS composition between the water phase and fuel phase.

## Conclusions

The bacterial surface is a heterogeneous structure with a complex chemical structure. Different functional groups present in bacterial macromolecules function at different times in binding to the surface and stabilizing the adhesive interaction. It was observed that for the AA7075-T6 surfaces, *P. putida* biofilm coverage was higher at the fuel–water interface, followed by the water phase and to a much lesser extent in the fuel phase. Also, the biofilm morphology is different in the fuel and water phase. *P. putida* formed localized cell aggregates or clusters in the fuel phase, adhered to the aluminum surface, whereas the cells in the aqueous phase were separate and homogeneously distributed across the aluminum coupon. EPS seems to play a role in protecting the cells and keeping them together, and its composition differs between the water phase and fuel phase.

Additionally, the adhesion of *P. putida* to the aluminum surfaces seems to be mediated by direct bonding of cell surface macromolecules on mature biofilms, where carboxylic and phosphoryl groups play a fundamental role, in particular, phosphoryl bonds from nucleic acids. This is a much stronger cell–surface bond, which makes biofilm removal more difficult once matured.

The use of enzyme DNase I to remove bacteria from the aluminum surface had an impact on both water and fuel phase biofilms, with the structure of the cells being disrupted at the aqueous phase but still attached to the aluminum coupon (i.e., disrupted dead biomass still remained on the surface). Because of irreversible chemical bonds between mature biofilm macromolecules and the surfaces, dead biomass on the aluminum surface will still remain despite biocide use. This could provide good regrowth conditions (e.g., nutrients and harbor sites for new bacteria) if not removed by physical means. Therefore, physical removal of the biofilms (mechanical cleaning) is as important as killing undesired biofilm organisms (e.g., using biocides or other antifouling agents) on the surfaces of aircraft materials such as aluminum alloy AA7075-T6. Cleaning protocols for the removal of undesired microbial contamination on aircraft surfaces should not only rely on biocides but also ensure a thorough physical removal of the biomass.
